# Phenomenological Inferences on the Kinetics of a Mechanically Activated Knoevenagel Condensation: Understanding the “Snowball” Kinetic Effect in Ball Milling

**DOI:** 10.3390/molecules24193600

**Published:** 2019-10-07

**Authors:** Maria Carta, Stuart L. James, Francesco Delogu

**Affiliations:** 1 Dipartimento di Ingegneria Meccanica, Chimica e dei Materiali, Università degli Studi di Cagliari, via Marengo 2, 09123 Cagliari, Italy; mcartak63@gmail.com; 2 School of Chemistry and Chemical Engineering, Queen’s University Belfast, David Keir Building, Stranmillis Road, Belfast BT9 5AG, UK; S.James@qub.ac.uk

**Keywords:** mechanochemistry, ball milling, Knoevenagel condensation, kinetics

## Abstract

We focus on understanding the kinetics of a mechanically activated Knoevenagel condensation conducted in a ball mill, that is characterized by sigmoidal kinetics and the formation of a rubber-like cohesive intermediate state coating the milling ball. The previously described experimental findings are explained using a phenomenological kinetic model. It is assumed that reactants transform into products already at the very first collision of the ball with the wall of the jar. The portion of reactants that are transformed into products during each oscillation is taken to be a fraction of the amount of material that is trapped between the ball and the wall of the jar. This quantity is greater when the reaction mixture transforms from its initial powder form to the rubber-like cohesive coating on the ball. Further, the amount of reactants processed in each collision varies proportionally with the total area of the layer coating the ball. The total area of this coating layer is predicted to vary with the third power of time, thus accounting for the observed dramatic increase of the reaction rate. Supporting experiments, performed using a polyvinyl acetate adhesive as a nonreactive but cohesive material, confirm that the coating around the ball grows with the third power of time.

## 1. Introduction

Mechanical processing by ball milling (BM) is a well established methodology in materials science [1,2]. Typically used to mix granular solids, reduce their particle size, refine their microstructure, and enhance their chemical reactivity [1,2], it has also been applied to chemical synthesis [3,4,5,6,7]. Physical and chemical transformations are activated and driven by the transfer of mechanical energy to the particulate. This activation takes place every time the particulate gets trapped between the surfaces of the milling tools (the ball and the jar walls) as they collide with each other [1,2,3,4,5,6,7]. Under the effect of the intense mechanical stresses generated during each collision, the granular solids undergo severe mechanical deformation [1,2,3,4,5,6,7]. Eventually, deformation results in the effective mixing of the solid phases on the microscopic scale, which is accompanied by the formation of extended interfaces between the reactants and the consequent enhancement of chemical reactivity [1,2,3,4,5,6,7].

The scenario mentioned above is particularly suitable to explain the transformations involving organic substances. Highly deformable, they readily undergo the shearing, compression, and folding processes that local mechanical stresses induce during collisions. The resulting intimate mixing affects the conditions under which chemical reactions occur [3,4,5,6,7] and can occasionally favour the formation of products different from those of thermally activated transformations [3,4,5,6,7].

In general, a close relationship between chemical reactivity and mechanical characteristics of the processed solids can be expected [1,2]. Accordingly, rheological behaviour of the particulate is expected to significantly affect the transformation kinetics. The recent kinetic study of a mechanically induced Knoevenagel condensation provided clear evidence for the importance of rheological effects [8].

**Scheme 1:**
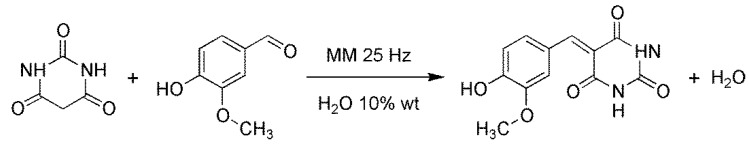
The mechanochemical Knoevenagel condensation of vanillin and barbituric acid.

The particular Knoevenagel condensation investigated was the model reaction between vanillin and barbituric acid shown in Scheme 1:

The reaction was successfully carried out under mechanical activation conditions for the first time in 2003 [9]. Since then, it has been investigated systematically under various milling conditions [10,11,12]. Experimental evidence clearly shows that the Knoevenagel condensation proceeds quantitatively in planetary and mixer ball mills, at a rate that is strongly influenced by milling parameters such as milling frequency, number of balls, and powder charge [10,11,12].

While these results confirm the appealing BM capability of activating organic transformations in the absence of solvent phases, additional evidence has been recently given concerning the role that the properties of processed substances can have on the transformation rate and the overall kinetic behaviour. In particular, it has been shown that the mechanical processing can induce a change in the physical form of the reaction mixture from a dry, loose powder to a cohesive, rubber-like state coating the ball [8]. This change takes place exactly during the sigmoidal increase in the transformation rate, thus suggesting that the rheological properties of reactants and products can affect the rate of the reaction, resulting in kinetics that differ dramatically from the simple first-order kinetics of the same reaction in solution [8].

Although such changes in the rheological characteristics have been reported in only a few cases [8,13,14], it is possible that they are quite general during mechanochemical reactions [15]. However, there is currently no mechanistic interpretation of how these rheological changes occur. In this work, we propose a phenomenological interpretation of the kinetic data collected in a previous investigation [8]. Our interpretation is based on a kinetic model that takes account of the statistical nature of BM. We support our kinetic analysis with an experimental investigation of the formation of cohesive rubber-like states in a suitably selected model system. In the following, we first recapitulate the conditions under which the Knoevenagel condensation between vanillin and barbituric acid was studied in the previous work [8]. We then describe the methods used here to perform and support the kinetic analysis.

## 2. Results

**Figure 1:**
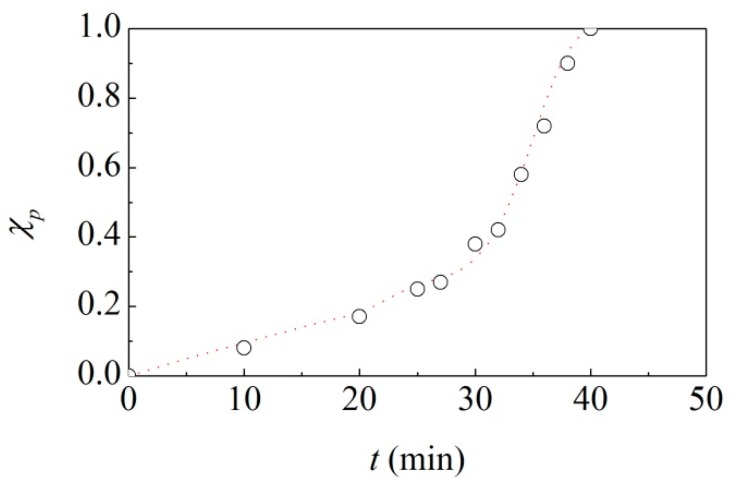
Kinetics of mechanochemical Knoevenagel condensation. The fraction of products, $\chi_{p}$, as a function of milling time, *t*. The best-fitted curve is shown.

The experimental and theoretical work carried out within the framework of the present investigation aims at explaining the kinetics of the mechanically activated Knoevenagel condensation between vanillin and barbituric acid described in the literature [8]. For convenience, the original kinetic dataset is reproduced in Figure 1.

The chemical conversion follows a sigmoidal trend characterized by a significant increase of the reaction rate approximately between 28 and 32 min of mechanical processing. The kinetic curve is quite different from the one observed for the same reaction carried out in solution, which corresponds to a simple first-order kinetics.

**Figure 2:**
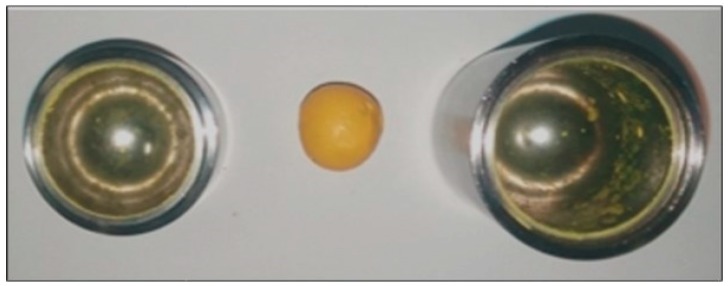
The cohesive state on the ball. Photograph of the steel milling ball with the cohesive rubber-like coating of the reaction mixture (centre) with the ball mill jar (left) and lid (right) almost free of material.

Hutchings et al. already noted that the sigmoidal kinetics can be related to “… the dramatic changes in the physical form of the reaction mixture as the reaction progressed. Specifically, the reaction mixture changed from a dry, free-flowing powder during the induction period to a cohesive rubber-like state that formed a robust coating around the ball during the sigmoidal increase in reaction rate, after which it returned again to a free-flowing powder…” [8]. A picture of the coated ball is given in Figure 2.

We show that such observation, in combination with a simplified kinetic modelling, can satisfactorily explain the experimental findings.

### 2.1. The Phenomenological Kinetic Model

Discussed in detail elsewhere [16,17,18], the model takes into account the fundamental features of BM. Accordingly, it assumes that:

(a) Only a small amount of powder is processed during each collision;
(b) The powder is trapped between the impacting milling tools during each collision with approximately stochastic dynamics;
(c) For any component of the powder mixture, the amount involved in each collision corresponds to the average composition of the powder charge;
(d) The chemical composition of the processed powder remains uniform during the entire mechanical treatment.

Additionally, it is reasonable to assume that (i) the mechanical stresses generated during each collision result in critical loading conditions (CLCs) only in a subvolume $V^{*}$ of the trapped powder, and that (ii) such subvolume remains approximately constant during the entire mechanical treatment. CLCs can be regarded as the mechanical loading conditions that generate mechanical stresses intense enough to cause the mechanical deformation required to activate a given transformation process. Thus, $V^{*}$ represents the volume of effectively processed powder. Both CLCs and $V^{*}$ can vary from process to process.

For a given transformation, the volume fraction of effectively processed powders is $\kappa =V^{*}/V$, where V is the total volume of powder inside the vial. Ideally, the powder charge can be divided into equal volume elements $V^{*}$ with the same probability of being processed in a given collision.

Once the first collision has occurred, the powder charge can be divided into two volume fractions, $\chi_{0}\left(1\right)$ and $\chi_{1}\left(1\right)$, of powders processed respectively zero and one times. These two fractions are equal to 1−κ and κ. As the second collision takes place, another volume fraction κ gets processed. Based on the model assumption that the powder charge remains always uniform, the volume fractions effectively processed zero, one, and two times become equal to $\chi_{0}\left(2\right)={\left(1-\kappa \right)}^{2}$, $\chi_{1}\left(2\right)=2\;\kappa \;\left(1-\kappa \right)$, and $\chi_{2}\left(2\right)=\kappa^{2}$.

The process continues as the number of collisions, n, increases. The volume fraction $\chi_{0}\left(n\right)$ of powder not yet processed after *n* collisions can be expressed as

(1) $$\chi_{0}\left(n+1\right)=\chi_{0}\left(n\right)-\kappa \;\chi_{0}\left(n\right)$$

This simply indicates that, during each collision, $\chi_{0}\left(n\right)$ decreases by the fraction $\kappa \;\chi_{0}\left(n\right)$. The corresponding expression for the fraction $\chi_{i}\left(n\right)$ of the powder processed i times after n collisions is more complicated, as its variation is governed by two different contributions. During the n-th collision, a fraction of the powder that has been processed i times, namely $\chi_{i}\left(n-1\right)$, is processed for the (i+1)-th time. This fraction contributes to $\chi_{i+1}\left(n\right)$, thus it must be subtracted from $\chi_{i}\left(n-1\right)$. On the other hand, the *n*-th collision will process a fraction of $\chi_{i-1}\left(n-1\right)$, equal to $\kappa \;\chi_{i-1}\left(n-1\right)$, for the *i*-th time, thus this amount must be added to $\chi_{i}\left(n-1\right)$. Therefore, after the n-th collision, the volume fraction of powder effectively processed *i* times is equal to

(2) $$\chi_{i}\left(n\right)=\chi_{i}\left(n-1\right)-\kappa \;\chi_{i}\left(n-1\right)+\kappa \;\chi_{i-1}\left(n-1\right)$$

As long as the volume fraction κ is small, i.e., κ << 1, which is the case in typical BM experiments, the discrete Equations (1) and (2) can be written in the continuous forms

(3) $$d\chi_{0}\left(n\right)=-\kappa \;\chi_{0}\left(n\right)\;dn$$

(4) $$d\chi_{i}\left(n\right)=-\kappa \;\chi_{i}\left(n\right)\;dn+\kappa \;\chi_{i-1}\left(n\right)\;dn$$

(5) $$\chi_{0}\left(n\right)=exp\left(-\kappa \;n\right)$$

whereas Equation (4) is solved by

(6) $$\chi_{i}\left(n\right)=\frac{{\left(\kappa \;n\right)}^{i}}{i!}exp\left(-\kappa \;n\right)$$

Equations (5) and (6), which satisfy the condition $\overset{\infty }{\underset{i=0}{\sum }}\chi_{i}\left(n\right)=1$, can be regarded as the fundamental equations that describe the kinetics of ball mill-activated transformations.

A kinetic curve with a simple exponential character can be obtained by assuming that the final product forms when the powder is processed above CLCs for the first time. Under this assumption, the volume fraction $\chi_{p}\left(n\right)$ of the product phase can be expressed as

(7) $$\chi_{p}\left(n\right)=1-exp\left(-\kappa \;n\right)$$

A sigmoid curve is obtained if the formation of the product requires that the powder undergoes CLCs twice. In this case,

(8) $$\chi_{p}\left(n\right)=1-\left(1+\kappa \;n\right)\;exp\left(-\kappa \;n\right).$$

If m CLCs are required, the resulting equation is more complicated, but retains the sigmoid shape and the volume fraction of the product can be expressed as

(9) $$\chi_{p}\left(n\right)=\overset{\infty }{\underset{i=m}{\sum }}\chi_{i}\left(n\right)$$

Similarly, if m CLCs are required to form an intermediate that evolves after p additional collisions, into a final product, the volume fraction of the intermediate can be expressed as

(10) $$\chi_{int}\left(n\right)=\overset{m+p}{\underset{i=m}{\sum }}\chi_{i}\left(n\right)$$

In all cases, the volume fraction of powder processed effectively during a collision, κ, can be regarded as a measure of the rate of the gradual mechanochemical transformation.

All of the model equations can be referred to mass fractions if density of reactants, intermediates if present, and products are taken into due account. In addition, under the assumption that the number of collisions, n, is proportional to time, t, all of the model equations reported above can be expressed as a function of time.

### 2.2. Analysis of Kinetic Data

The capability of mechanical processing by BM of inducing the Knoevenagel reaction raises a crucial question for its kinetic analysis. Is the reaction activated by the very first impact? Or, perhaps, a given fraction of powder must be hit twice to undergo the chemical transformation? At present, we do not have direct evidence concerning the point. Under these circumstances we make the simplest possible assumption that allows kinetic modelling. Accordingly, we assume that the Knoevenagel condensation takes place, in a small fraction of the powder trapped between ball and vial, when the powder has undergone CLCs once. If the rate coefficient, k, accounting for the transformation rate remained constant, we could equal it to κ and try to use the equivalent of Equation (7) referred to time to describe the reaction kinetics. However, a constant k does not seem to be the case for the observed transformation.

The rate coefficient k measures the volume fraction processed effectively during a single collision. Therefore, it is proportional to the volume of material subjected to CLCs. In turn, the volume of material subjected to CLCs can be expected to scale with the amount of material that can be effectively trapped between the surfaces of milling tools during the collision.

The efficiency of trapping can vary greatly depending on the nature of the processed material. Trapping loose powder is quite difficult because of the intrinsic dynamics of the granular body inside the reactor. Thus, it can be expected, and it is also generally observed [16,17,18], that only a very small fraction of the powder charge is effectively processed in individual collisions. In contrast, a greater trapping efficiency can be expected when the processed material coats the milling tools. Indeed, a significant fraction of the whole powder charge inside the reactor will be certainly loaded by milling tools under such circumstances.

According to the experimental findings regarding the Knoevenagel condensation, the material inside the reactor forms a coating around the milling ball. Such coating forms gradually as a consequence of the ball impacting and rolling inside the reactor and we can suppose that it makes k increase with time. In particular, we can expect that the coating forms at a rate proportional to the total surface that can be coated. It follows that the coating rate can be expressed as

(11) $$\frac{dv}{dt}=4\;\pi \;r^{2}\frac{dr}{dt},$$

where v is the volume of material forming the coating layer around the milling ball and r is the radius of the coated ball. Now, we can suppose that the rate of radius increase remains constant. This means that

(12) $$\frac{dr}{dt}\sim c.$$

Thus,

(13) $$r=c\;t+r_{0}$$

where $r_{0}$ is the radius of the uncoated ball. Once we substitute Equation (13) in Equation (11) and integrate Equation (11), the result is

(14) $$v=\;\frac{4}{3}\;\pi \;{\left(c\;t+r_{0}\right)}^{3}-\frac{4}{3}\;\pi \;r_{0}^{3}$$

It follows that the volume of the coated ball is

(15) $$v_{ball}=\;\frac{4}{3}\;\pi \;{\left(c\;t+r_{0}\right)}^{3}$$

If we assume that the rate coefficient k is proportional to the volume $v_{ball}$ of the coated ball, then

(16) $$k=\frac{A}{V}\;\frac{4}{3}\;\pi \;{\left(c\;t+r_{0}\right)}^{3}$$

where V is the total volume of powder inside the reactor and A is a proportionality constant. Thus, to a first, rough approximation, we can expect that k exhibits a cubic dependence on time.

It is also worth noting that experimental findings suggest that k increases definitely only after about 20 min of mechanical processing. Therefore, it seems reasonable to assume that the material starts coating the ball only after an induction period $t_{0}$.

The above-mentioned observations can be summarized as follows:

(17) $$k\left(t\right)=k_{0},\;t<t_{0}$$

(18) $$k\left(t\right)=k_{0}+\frac{A}{V}\;\frac{4}{3}\;\pi \;{\left(c\;t+r_{0}\right)}^{3},\;t\geq t_{0}$$

Here, $k_{0}$ is the apparent rate constant of the transformation for times shorter than the induction period $t_{0}$, when the material is still unable to stick to the ball surface and form a stable coating. For times longer than $t_{0}$, the apparent rate constant k is affected by the additional contribution related to coating.

The variation of k with time does not allow any analytical description of the kinetic curve. However, it is still possible to write the equations as a function of time and solve them numerically.

(19) $$d\chi_{0}\left(t\right)=-k\left(t\right)\;\chi_{0}\left(t\right)\;dt$$

(20) $$d\chi_{i}\left(t\right)=-k\left(t\right)\;\chi_{i}\left(t\right)\;dt+k\left(t\right)\;\chi_{i-1}\left(t\right)\;dt$$

Equivalent to Equations (3) and (4), Equations (19) and (20) provide a general description of the transformation kinetics. A best-fitting procedure allows us to find the expression of the kinetic curve and the spectrum of k(t). values associated.

**Figure 3:**
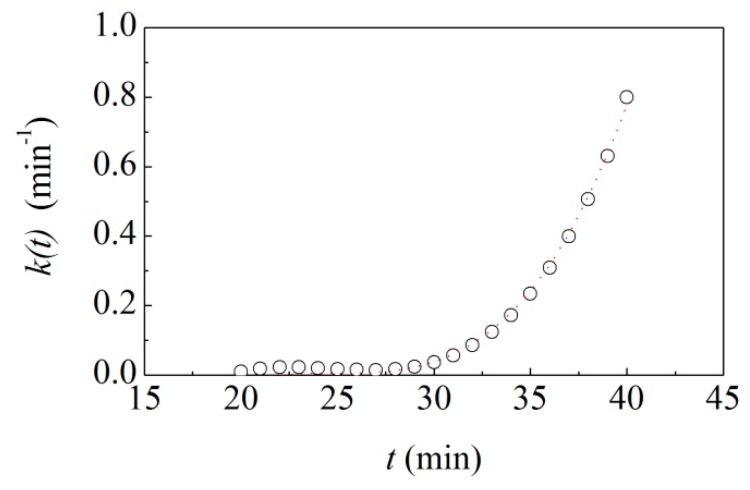
The variation of the rate coefficient. The rate coefficient, k(t), as a function of time *t*. The best-fitted curve is shown.

The hypothesis that the reaction activation requires that powder undergoes CLCs only once allows a very good fitting of the experimental data. As shown in Figure 1, the resulting kinetic curve perfectly interpolates the experimental points. Such kinetic curve is obtained allowing k(t) to vary in time as shown in Figure 3. It can be seen that, starting from a relatively low value, k(t) progressively increases up to about 0.8 min^−1^.

Now, it is worth noting that Equation (18) satisfactorily describes the variation of k(t). The best-fitted curve almost overlaps the k(t) estimates, providing significant support to the theoretical model proposed. In particular, it seems definitely reasonable to expect that cohesive states develop gradually under the effect of adhesive forces between the coated ball and the material present in the vial. The mechanism is analogous to that governing the formation of snowballs. Accordingly, the rate of adhesion to the coating depends on the amount of material involved in the coating itself and the adhesion process proceeds autocatalytically until all the material inside the vial has been collected or other competing disaggregation processes intervene to limit the growth of the coating layer.

The satisfactory best-fitting allowed obtaining reliable estimates for the different unknown quantities in Equation (18). In particular, it suggests an induction period, $t_{0}$, about 27 min long and, for times shorter than 27 min (not shown for clarity in Figure 3), an initial value, $k_{0}$, of about 0.01 min^−1^ for the rate coefficient. The rate of radius increase, c, is approximately equal to 0.1 mm min^−1^, a value that properly accounts for the rapid formation of the coating layer.

The results discussed heretofore suggest that the kinetic modelling can suitably explain the experimental findings concerning the Knoevenagel condensation between vanillin and barbituric acid carried out under mechanical activation conditions [8]. Specifically, it seems that the simplest possible kinetic assumption that the reaction occurs in a fraction of the processed material already at the first impact, combined with the hypothesis that the rate of coating formation is proportional to the total surface that can be coated, leads to kinetic equations able to reproduce the experimental data quite satisfactorily.

However, there are at least two issues that deserve further investigation in the attempt of gaining a deeper insight into the behaviour of the processed material and validating, as far as possible, the kinetic model proposed. On the one hand, it is worth investigating whether or not cohesive, rubber-like states can form under the effects of a mechanical action different from the one imparted to milling balls by the Retsch MM400 shaker-type ball mill used in previous work [8]. On the other, it is highly desirable to obtain direct evidence concerning the formation kinetics of the coating layer around the milling ball in order to support the hypothesis underlying Equations (14)–(16).

To this aim, we expressly designed and carried out experiments involving a different ball mill and a different adhesive material. Specifically, in view of the number of preliminary runs to be done under different conditions, we used polyvinyl acetate glue. In addition, we equipped the ball mill with the sensors needed to monitor the milling conditions and obtain in situ indirect information on the rheological behaviour of the material inside the vial. Details are given in the following.

### 2.3. Supporting Experiments

Experiments were performed using a SPEX Mixer/Mill 8000. It was chosen because of the different mechanical action with respect to the Retsch MM400 ball mill. While the latter makes the vials oscillate on the horizontal plane with a simple harmonic motion, the SPEX Mixer/Mill 8000 swings the vial along a three-dimensional trajectory that combines a vertical harmonic motion with synchronous oscillations on the horizontal plane. It follows that the vial movement results in a more efficient stirring of powder and a wider volume spanned by the milling ball. In addition, we also used a SPEX vial with a flat base, which makes ball trajectories inside the vial more complicated and impacts on the vial walls more energetically.

The use of a SPEX Mixer/Mill 8000 has another advantage. The milling dynamics have been thoroughly studied under the most diverse experimental conditions [19,20,21], which provided significant help in the planning of experiments and the interpretation of their results. Following previous work [19], a single milling ball was used. Furthermore, the ball mill was equipped with a piezoelectric transducer in order to monitor the occurrence of impacts between the ball and the vial walls. The sensor was placed on the bottom end of the vial and connected to a computer to record the sequence of electric signals generated by impacts. The polymer, initially in the liquid phase, was added as a partially dried powder.

**Figure 4:**
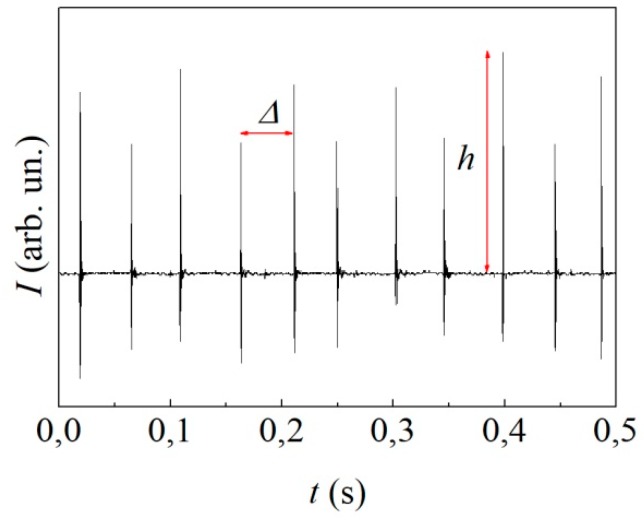
A sequence of signals generated by the piezoelectric sensor. The period between consecutive signals, Δ, and the height of signals, h, utilized as a measure of intensity are indicated.

A short sequence of signals generated by the piezoelectric sensor is shown in Figure 4. Signals are regularly spaced, thus indicating that the ball underwent regular dynamics. Two impacts per vial cycle were detected, which corresponds to an impact frequency of about 23.2 Hz.

We recorded long sequences of signals to gain information on the ball dynamics on long time-scales. Although more complex analyses can be performed, the distance between two consecutive signals and the intensity of signals allow sufficient insight in this respect. The former gives information on the periodicity of the ball motion, with relatively short values pointing out the occurrence of irregular trajectories with multiple rebounds on the vial base and cylindrical wall. The latter measures the energy dissipation at collisions, with a decrease of signal intensity for less energetic collisions.

**Figure 5:**
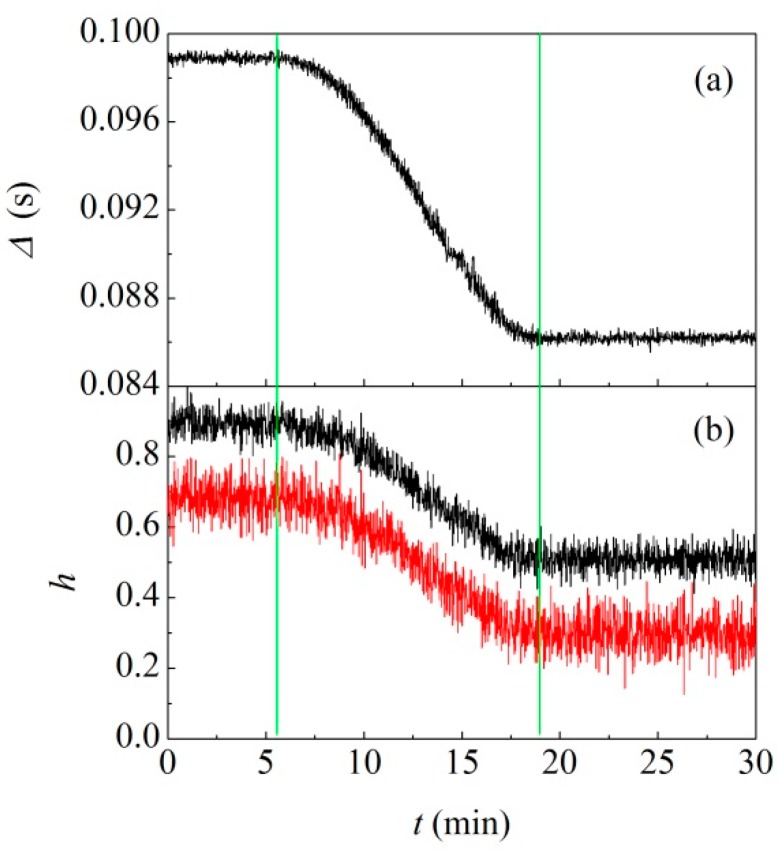
The transition in milling dynamics. (**a**) The distance, Δ, between consecutive signals and (**b**) the relative intensity, h, of signals due to impacts on the bottom base (black) and the vial cap (red). The two vertical green lines mark the transition period.

The distance between two consecutive signals, Δ, is shown in Figure 5a as a function of time, t.

It can be seen that Δ remains approximately constant during the first 6 min of BM. Then, it decreases slowly for other 13 min, after which it keeps the constant value of 23.2 Hz, twice the milling frequency. Overall, data indicate that the ball never undergoes chaotic dynamics. However, after about 20 min, there is a clear transition to an extremely regular behaviour. Based on the impact frequency as well as on previous work on other systems [19,20,21], it can be inferred that the ball has reached the most regular possible dynamics. Accordingly, it simply travels between the opposite bases undergoing almost perfectly inelastic collisions.

The relative intensity of signals, h, normalized to the maximum h value observed, is plotted in Figure 5b. Two distinct sequences of h values were detected, simply due to the position of the piezoelectric sensor. Being placed externally on the bottom end of the vial, it recorded with higher intensity the impacts occurring on the bottom base. Those occurring on the vial cap were much less intense, also because of the O-ring that, while assuring a good sealing of the vial, dampened any vibration. Stronger and weaker signals exhibited a similar variation with time. They initially kept an approximately constant intensity. Then, the intensity decreased progressively, and significantly, until a new constant value was reached.

**Figure 6:**
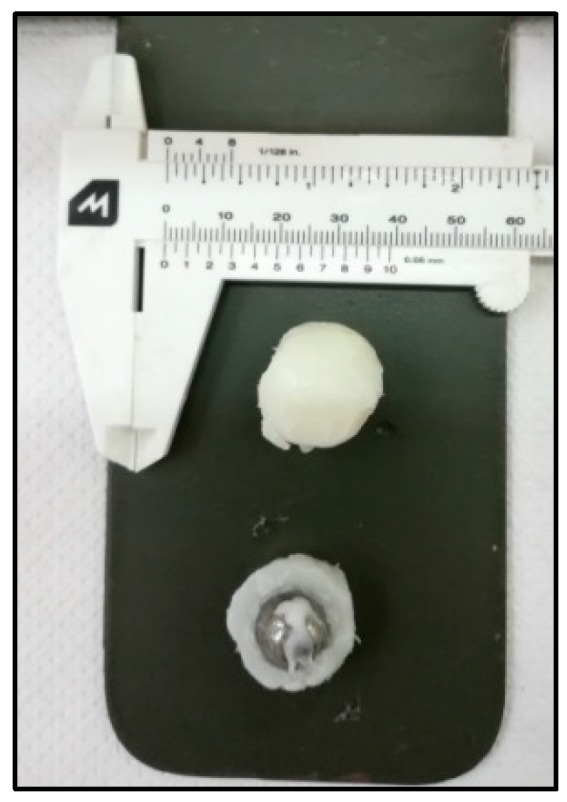
Milling balls after 24 min of milling. A ball completely coated (top) and a ball with the coating partially removed with the help of a sharp cutter.

The variation of h is substantially synchronous with the variation of the distance Δ between consecutive impacts. This strongly suggests that something happened to the ball after about 6 min of mechanical processing, as evident from the direct inspection of the ball after 3 min and after 24 min of milling. Before the transition in the ball dynamics occurred, the ball was still quite clean. Conversely, once the ball had reached the new dynamics, the ball was completely coated with material as shown by the picture in Figure 6.

**Figure 7:**
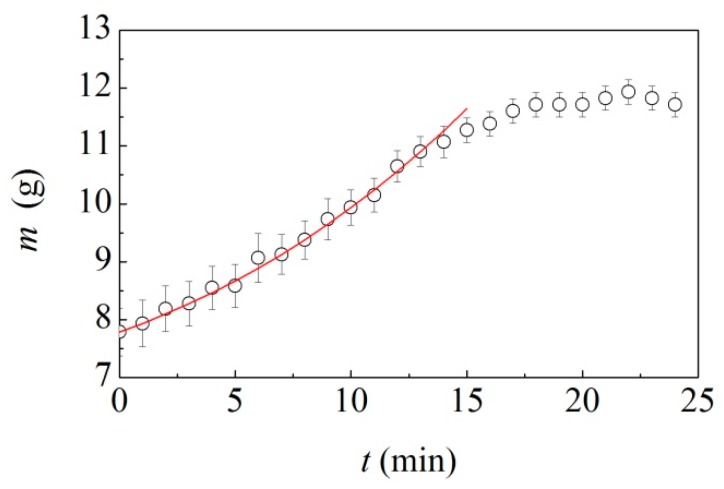
The kinetics of adhesion. The mass of the ball, m, as a function of time, t. Data have been averaged over three independent experiments. The best-fitted cubic curve is shown. The zero in the abscissa axis corresponds to the fifth minute of milling.

Based on the evidence mentioned above, we opened regularly the vial to weigh the ball during the transition period starting from 5 min of milling. We repeated the experiments three times to suitably account for experimental uncertainties. At the beginning of BM, the material inside the jar kept its powder form, although it showed tendency to aggregate. As the coating layer around the ball began to form, part of the powder adhered to the rubber-like cohesive state. Generally, it could be recognized and easily separated from the coated ball. In the later BM stages, the material not yet involved in the formation of the cohesive layer around the ball also formed irregular coatings on the vial. Eventually, all the material was consumed in the coating layer. The results are shown in Figure 7, where the mass of the coated ball, m, is plotted as a function of time, t. Data arranged according to a concave up, increasing curve.

Assuming that the mechanical processing does not change the density of the adhesive material, so that it keeps constant throughout the milling, Equation (14) can be readily utilized for expressing the mass of the coated ball. It can be rewritten as

(21) $$m=\;\frac{4}{3}\;\pi \;\rho_{a}\;\left[{\left(c\;t+r_{0}\right)}^{3}-r_{0}^{3}\right]+\frac{4}{3}\;\pi \;\rho_{b}r_{0}^{3}$$

where $\rho_{a}$ and $\rho_{b}$ are the densities of adhesive and ball, equal to 1.1 and 7.85 g cm^−3^ respectively. We used Equation (21) to interpolate the experimental data in Figure 7. It can be seen that it works quite well. Thus, it can be reasonably deduced that the coating around the ball grows proportionally to the total surface of the coated layer and that the thickness of the coated layer varies linearly with time.

## 3. Discussion

The results presented heretofore provide significant insight into the main factors and processes underlying the kinetics of the mechanically activated Knoevenagel condensation between vanillin and barbituric acid investigated in previous work [8], and help clarify the interplay between the degree of chemical conversion and the rheological properties of the substances subjected to BM.

The first aspect to note concerns the shape of the kinetic curve that is suggested by experimental points shown in Figure 1. As already observed by Hutchings et al. [8], the chemical conversion is initially relatively slow and only subsequently its rate undergoes a marked increase that can be related to a modification of the rheological properties of the processed chemicals. The kinetic analysis we have carried out throws new light on both these issues.

First, it is worth noting that the kinetic model describes satisfactorily the initial portion of the kinetic curve without the need for a time dependent rate coefficient k. Accordingly, experimental data can be best-fitted by Equation (7) up to a milling time of about 27 min, which corresponds to the induction period *t*_0_. Therefore, the initial portion of the kinetic curve that best fits the data in Figure 1 has the simple exponential character resulting from the assumption that the final product forms when the powder is processed above CLCs for the first time. In this regard, it is worth remembering that the kinetic model relates the chemical conversion under BM conditions to individual impacts, but makes no microscopic hypothesis on the kinetic processes and mechanisms that rule the transformation during the impact. It follows that the shape of the kinetic curve is not determined by chemistry, but mostly by the number of times the powder has to be processed above CLCs to trigger the chemical reaction.

Second, the increase in transformation rate observed in the second stage of the Knoevenagel condensation has been correctly ascribed to the modification of the rheological behaviour of the processed substances and, in particular, to the formation of rubber-like, cohesive states around the milling ball. There exists a simple phenomenological explanation for the fact that the formation of a coating layer around the milling ball gives rise to a rate increase of chemical conversion. The amount of material processed above CLCs is, somehow, proportional to the amount of material that the ball traps during individual impacts. In turn, the latter quantity depends, somehow, on the size of the milling ball. In the presence of adhesion processes, a layer of adhesive material coats the ball and its thickness increases with time, making the coated ball bigger and bigger. It follows that the coated ball can trap an ever-increasing amount of material and the resulting behaviour mimics an autocatalytic chemical process.

In the absence of a proper kinetic modelling, it is quite hard to go beyond the phenomenological explanation mentioned above. However, we used a suitable conceptual framework to describe the fundamentals of BM and our kinetic model can be used to quantitatively analyse the kinetics of the Knoevenagel condensation between vanillin and barbituric acid studied in previous work [8]. Briefly, we utilized a general form of model equations, namely Equations (19) and (20), involving a time-dependent rate coefficient and we performed a direct inversion procedure to best fit the experimental data in Figure 1 and extract the best-fitted values of the time-dependent rate coefficient k(t). Plotted in Figure 3, the obtained best-fitted values clearly show that *k*(*t*) increases with time, providing a first, generic support to the hypothesis that the apparent autocatalytic behaviour is simply the result of an increased trapping efficiency of the coated ball. A trivial data analysis suggests that k(t) exhibits a cubic dependence on time. As shown by Equations (15) and (16), this is exactly the time dependence expected for k(t) based on the assumption that the amount of material trapped by the ball during individual impacts is proportional to the ball size. Moreover, Equation (18) is able to best fit the spectrum of k(t) values. Thus, we have indirect information on the formation rate of the rubber-like cohesive states. It appears that the layer thickness of the adhesive material grows at about 0.1 mm min^-1^, which is a reasonable value for the adhesion processes observed in previous work concerning the Knoevenagel condensation [8].

Starting from the results of the kinetic analysis discussed so far, we have gone a step further. In particular, we carried out new experiments to verify the occurrence of adhesion processes in a ball mill with a different mechanical action, ascertain their time dependence and investigate the underlying kinetics in situ. To this aim, we processed suitable amounts of polyvinyl acetate glue in a SPEX Mixer/Mill 8000 in the presence of a single milling ball and at a relatively low milling frequency. The experimental findings give clear indications regarding both the reliability of our methodological approach to monitor the milling dynamics and the rate of the adhesion processes. In particular, a piezoelectric transducer on the bottom end of the vial suffices to point out the occurrence of a transition in the impact conditions during the BM of the polyvinyl acetate glue. The gradual modification of the impact conditions involves a regularization of the ball trajectories between the opposite vial bases and can be ascribed to the formation of a coating layer around the milling ball. We measured the thickness of the coating layer and we found that it increases with time according to the third power of time. This means that the rate of coating formation is proportional to the coating surface and that the coating thickness increases linearly with time. Therefore, the experimental findings justify a posteriori the hypothesis made initially to carry out the kinetic analysis. Overall, this provides direct support to the kinetic analysis carried out on the data describing the kinetics of the Knoevenagel condensation and it suggests that our approach can be extended to similar cases.

## 4. Materials and Methods

For convenience, we report the experimental conditions that, in previous work [8], allowed observing the formation of cohesive, rubber-like states during the Knoevenagel condensation between vanillin and barbituric acid. Chemicals with >98 % purity were purchased from Sigma Aldrich UK and were used as received, unless indicated. BM experiments were carried out using a Retsch MM400 mixer mill. Vanillin (0.29 g, 1.9 mmol), barbituric acid (0.24 g, 1.9 mmol), the required amount of water, and a steel grinding ball (13.6 g) were added to a 25 cm^3^ stainless steel vial. The vial was shaken at 25 Hz for the required amount of time. The product was a powder, yellow if the reaction was incomplete, orange if complete [8].

The new experiments aiming at explaining the formation of rubber-like cohesive states were carried out using polyvinyl acetate in the form of commercial wood glue. Preliminary tests at room temperature showed that the liquid exhibited relatively low viscosity. Viscosity progressively increased as the glue hardened, which took place on the time scale of about 2 h.

We added 5 mL of the liquid adhesive to the hardened steel vial of a SPEX Mixer/Mill 8000 together with a stainless steel ball with diameter of 0.62 cm and mass of about 7.8 g. Once sealed, the vial was clamped on the mill and equipped with a piezoelectric transducer on the bottom end to detect the collisions between ball and container. Then, the mill was operated at 11.6 Hz, which is the lowest milling frequency that the mill can bear to keep the vial swing regular.

We interrupted the milling and opened the vial at regular time intervals to take the ball coated with adhesive material, and weighed it using a laboratory precision balance able to read four decimal places. Every time, vial and ball were perfectly cleaned, a further 5 mL of adhesive was put inside the vial, and the milling restarted up to the subsequent time interval selected.

## 5. Conclusions

The use of a kinetic model able to take into due account the intrinsic statistical nature of BM allows the phenomenological interpretation of the sigmoidal kinetics exhibited by the Knoevenagel condensation carried out under mechanical activation conditions. The observed kinetics are satisfactorily described assuming that reactants subjected to CLCs at least once transform into products and that the apparent rate constant increases with time after an induction period. The increase of the apparent rate constant with time is connected with the formation of a stable coating around the ball. As the processed material sticks to the ball, the amount of reactants subjected to the necessary CLCs per collision increases. The kinetic analysis suggests that the apparent rate constant varies with the third power of time and support experiments, performed using a polyvinyl acetate adhesive, confirm such time dependence. Specifically, the mass coating the ball increases according to third-power kinetics. This supports the kinetic analysis carried out and provides a general conceptual framework to investigate similar case studies.
